# Development of 3D Printed Biodegradable Mesh with Antimicrobial Properties for Pelvic Organ Prolapse

**DOI:** 10.3390/polym14040763

**Published:** 2022-02-16

**Authors:** Jiongyu Ren, Rebecca Murray, Cynthia S. Wong, Jilong Qin, Michael Chen, Makrina Totsika, Andrew D. Riddell, Andrea Warwick, Nicholas Rukin, Maria A. Woodruff

**Affiliations:** 1 Centre for Biomedical Technologies, Queensland University of Technology (QUT), Brisbane, QLD 4000, Australia; edward.ren@qut.edu.au (J.R.); m66.chen@hdr.qut.edu.au (M.C.); 2 School of Mechanical, Medical and Process Engineering, Queensland University of Technology (QUT), Brisbane, QLD 4000, Australia; 3 Herston Biofabrication Institute, Metro North Health, Brisbane, QLD 4029, Australia; becca.murray@health.qld.gov.au (R.M.); nicholas.rukin@health.qld.gov.au (N.R.); 4 Australian Institute for Bioengineering and Nanotechnology, The University of Queensland, Brisbane, QLD 4072, Australia; 5 Redcliffe Hospital, Metro North Health, Redcliffe, QLD 4020, Australia; adriddell@hotmail.com (A.D.R.); mda99amw@yahoo.com (A.W.); 6 Aikenhead Centre for Medical Discovery (ACMD), St Vincent’s Hospital, Melbourne, VIC 3065, Australia; c_wong32@hotmail.com; 7 Centre for Immunology and Infection Control, School of Biomedical Sciences, Queensland University of Technology, Brisbane, QLD 4000, Australia; jilong.qin@qut.edu.au (J.Q.); makrina.totsika@qut.edu.au (M.T.); 8 Northside Clinical Unit, School of Clinical Medicine, The University of Queensland, Brisbane, QLD 4072, Australia

**Keywords:** pelvic organ prolapse, controllable degradation rate, polycaprolactone, polyethylene glycol, antibacterial, biocompatible

## Abstract

To address the increasing demand for safe and effective treatment options for pelvic organ prolapse (POP) due to the worldwide ban of the traditional polypropylene meshes, this study introduced degradable polycaprolactone (PCL)/polyethylene glycol (PEG) composite meshes fabricated with melt-electrowriting (MEW). Two PCL/PEG mesh groups: 90:10 and 75:25 (PCL:PEG, wt%) were fabricated and characterized for their degradation rate and mechanical properties, with PCL meshes used as a control. The PCL/PEG composites showed controllable degradation rates by adjusting the PEG content and produced mechanical properties, such as maximal forces, that were higher than PCL alone. The antibacterial properties of the meshes were elicited by coating them with a commonly used antibiotic: azithromycin. Two dosage levels were used for the coating: 0.5 mg and 1 mg per mesh, and both dosage levels were found to be effective in suppressing the growth of *S. aureus* bacteria. The biocompatibility of the meshes was assessed using human immortalized adipose derived mesenchymal stem cells (hMSC). In vitro assays were used to assess the cell viability (LIVE/DEAD assay), cell metabolic activity (alamarBlue assay) and cell morphology on the meshes (fluorescent and electron microscopy). The cell attachment was found to decrease with increased PEG content. The freshly drug-coated meshes showed signs of cytotoxicity during the cell study process. However, when pre-released for 14 days in phosphate buffered saline, the initial delay in cell attachment on the drug-coated mesh groups showed full recovery at the 14-day cell culture time point. These results indicated that the PCL/PEG meshes with antibiotics coating will be an effective anti-infectious device when first implanted into the patients, and, after about 2 weeks of drug release, the mesh will be supporting cell attachment and proliferation. These meshes demonstrated a potential effective treatment option for POP that may circumvent the issues related to the traditional polypropylene meshes.

## 1. Introduction

Pelvic organ prolapse (POP) and incontinence are common and significant problems for women. It has been estimated that half of all women will have either symptoms or signs of prolapse after the menopause. Studies suggest that 10–15% of women in developed countries will undergo surgery for prolapse during their lifetime [1]. Patients with symptoms of urinary and faecal incontinence and POP, were commonly treated via implantation of polypropylene (PP) pelvic meshes [2,3]. The pelvic mesh was expected to reinforce the pelvic organ, as well as prevent recurrence of the symptoms. Data from the Therapeutic Goods Administration (TGA) showed that 151,000 meshes have been implanted in Australia since 1998 [4] and 3.7 million world-wide between 2005 and 2013 [5]. However, complications of pelvic mesh implantation such as erosion into vagina, infection, pain and discomfort were occurring, with some patients requiring further surgery [5]. The high rates of complication prompted the Food and Drug Administration (FDA) to issue 2 warnings against the use of certain pelvic meshes [6], leading to a worldwide withdrawal of a number of products, such as Gynecare Prolift^®^, Prolift+M™, Prosima™, and Anterior Pinnacle™ kits [7]. New Zealand was the first country in the world to ban the use of transvaginal POP mesh products in 2017 and followed by UK in 2018 [8]. According to TGA data, in 2018 Australia cancelled the approval of all mesh devices placed through the vagina for POP and required all mesh devices to be subject to a comprehensive review before being supplied. The worldwide bans have created a significant unmet clinical need of providing women with good treatment options and viable therapeutics.

Meshes used in the past were predominantly made from polypropylene, a non-degradable polymer. The risk of erosion was in part due to the trans-vaginal placement of mesh and the difference in mechanical properties of the mesh compared to natural tissue. Apart from erosion, another major issue with the implanted meshes is that they can become infected. Studies have shown high rates of infection in meshes whereby two-thirds of these patients often develop infection 2–4 years following implantation [9]. Bacteria that are commonly found in the infected meshes are *Escherichia coli* and *Staphylococcus aureus* and 31% of patients swabbed presented with multibacterial infections consisting of *P. mirabilis*, *E. coli*, *Staphylococcus*, *Streptococcus* and *Enterococcus* [10]. 

Prior to the worldwide ban, the commercially available pelvic meshes were generally created via knitting or weaving techniques [11,12]. These polypropylene meshes were designed to exhibit high tensile strength to support the pelvis. The stiffness of commercial meshes ranges from 11 N/mm for SmartMesh to 28 N/mm for Gynemesh [11]. However, the stiffness of the meshes also contributed to the failure due to their mismatch in mechanical properties compared to the vaginal tissue [11].

Additive manufacturing techniques are widely used in tissue engineering and regenerative medicine to fabricate three-dimensional (3D) printed scaffolds or meshes. One technique that provides a high degree of control in scaffold fabrication is melt-electrowriting (MEW), which is well established in our lab [13,14]. In this study, biodegradable polymers, polycaprolactone (PCL) and polyethylene glycol (PEG), both FDA approved for medical devices, were used to fabricate composite meshes. 

In this project, we aim to fabricate resorbable mesh with tailorable degradation rate and antibacterial properties as a potential solution to the clinically used polypropylene meshes. It is anticipated that creating a tissue-substitute that is anti-bacterial and imparts compatible biological and mechanical properties such as tensile strength and stiffness can address current limitations of the pelvic mesh.

## 2. Materials and Methods

**Figure 1:**
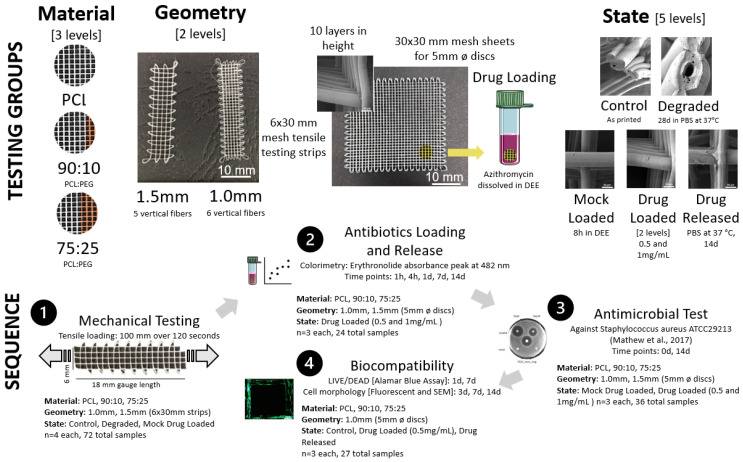
The testing groups showing details on the sample material (PCL, 90:10, 75:25), geometry in terms of spacing and sample size, and state (**top**). The testing sequence is also shown with details of testing type, sample groupings and sample size (**bottom**).

Testing was performed to investigate the effect of mesh material (PCL, PCL/PEG 90:10, PCL/PEG 75:25) mesh geometry via cross hatch spacing (1 mm vs. 1.5 mm) and mesh state (control, degraded, mock loaded, drug loaded (0.5 and 1 mg/mL), drug released). Various groupings were tested mechanically via tensile testing for antibiotic loading and release, antimicrobial test and biocompatibility (Figure 1). 

### 2.1. PCL/PEG Composite Preparation

Medical grade polycaprolactone (mPCL) (Purasorb^®^ PC 12, Corbion Purac Biomaterials, The Netherlands) and PEG (Mw 20,000, Sigma-Aldrich, Australia) were dissolved in chloroform in weight ratios of 90:10 and 75:25. The polymer composites were mixed for 8 h and left in the fume hood until complete evaporation of solvents. The PCL/PEG 90:10 and PCL/PEG 75:25 were used for mesh printing with an in-house built melt-electrowriting (MEW) device.

### 2.2. Melt-Electrowriting of PCL and PCL/PEG Meshes

The MEW device produces the meshes using an applied pressure to extrude a molten polymer through a positively charged nozzle onto a grounded motorized collector plate according to the parameters detailed in Table 1 below. The collector plate translates in x and y directions controlled by a Gcode using computer programming. The details of the MEW process can be found in our previous study [13]. PCL and PCL/PEG meshes were produced with a 90° cross-hatched fibre deposition in 10-layer high sheets (30 × 6 mm and 30 × 30 mm). The meshes were fabricated with a fibre spacing of 1 mm and 1.5 mm with the groups of meshes used in this study summarized in Figure 1 and the MEW printing parameters used are shown in Table 1.

### 2.3. Mesh Degradation

#### 2.3.1. Physiological Condition Degradation

Three mesh samples from each group were weighed and immersed in 10 mL phosphate-buffered saline (PBS). The samples in PBS solution were placed on a rotator in an oven and the temperature was kept at 37 °C for 28 days. The meshes were assessed for mass loss at 1 day, 7 d, 14 d, 21 d and 28 d time points.

#### 2.3.2. Accelerated Degradation

Three mesh samples of each group were weighed and immersed in 10 mL 5 M NaOH at 37 °C until the samples became irretrievable as reported previously [15]. The meshes were assessed for mass loss at 1 h, 3 h, 6 h, 10 h, 1 d, 3 d and 7 d.

### 2.4. Mechanical Testing

The 30 × 6 mm mesh strips were used for the tensile testing with a Tytron 250 Microforce Testing System (MTS Systems Corp., Minnesota, MN, USA) using a load cell (Model 661. 11B-02, 2.5 mN resolution). Test samples consisted of two mesh geometries (1.0 mm and 1.5 mm) and three materials (PCL, 90:10, 75:25), resulting in 18 groups (*n* = 4 each). All samples were tested using an 18 mm gauge length to stretch for 100 mm over 120 s. Force-displacement data were obtained from the tests and the stress-strain curves were obtained by calculating the stress (σ) from the force divided by the average cross-sectional area of the meshes (Equation (1)) and strain (ε) by normalising the initial displacement (Equation (2)). The test cross sectional area was approximated as the sum of the circular longitudinal fibres, where the 1.0 mm meshes had 60 fibres (6 across and 10 layers) and the 1.5 mm mesh had 50 fibres (5 across × 10 layers), and the fibre diameter was taken as the average of 5 measurements from SEM images for each material (PCL = 55.6 µm, 90:10 = 66.4 µm, 75:25 = 51.4 µm). Elastic modulus and yield strength were calculated from a 0.2% offset linear best fit line between 30–70% strain. Ultimate tensile strength (UTS) is the maximum stress the mesh endured, and maximum force is defined as the maximum force (N) per 1 cm cross-sectional width (enables comparison to other mesh types).

(1) $$\sigma =\frac{F}{A}=\frac{F}{\pi {\left(\frac{Ø}{2}\right)}^{2}ln}$$

where *F* is the measured force, *A* is the mesh cross sectional area, Ø is the diameter of fibres, *l* is the number of mesh layers and n is the number of vertical fibres (5 for 1.5 mm meshes and 6 for 1.0 mm meshes).

(2) $$\varepsilon =\frac{\Delta L}{L}=\frac{d-d_{o}}{L}$$

where ∆*L* is the change in sample length, *L* is the gauge length of samples (18 mm), *d* is the measured displacement and $d_{o}$ is the initial displacement value.

### 2.5. Antibiotics Loading and Release Profile

Azithromycin, a broad-spectrum antibiotic, was coated onto the meshes and assessed for its antibacterial potency.

#### 2.5.1. Antibiotics Loading

Azithromycin was loaded onto the meshes using a method adopted from a previous study [16]. Briefly, the 30 × 30 mm mesh sheets were cut into disks of 5 mm in diameter and placed in 1 mL flat bottom centrifuge tubes with screwable caps. The drug loading solutions were prepared by dissolving 0.5 mg and 1 mg of azithromycin in 100 μL of diethyl ether (DEE), and the mesh disks were incubated with the loading solutions for 8 h at room temperature with mild agitation. Following the incubation, the meshes were air dried in a fume hood for complete DEE evaporation and the drug loaded meshes were kept at −20 °C for further analysis. 

#### 2.5.2. Antibiotics Loading Efficiency

Azithromycin concentration was measured by colorimetry by mixing the azithromycin with 43% sulfuric acid solution [16]. Erythronolide, the hydrolysis degrative product of azithromycin, exhibits an absorbance peak at 482 nm, and this was used to quantify the concentration of azithromycin based on the absorbance intensity [17]. The azithromycin standard solutions were prepared by incubating the drug powder at concentrations of 1, 5, 10, 15, 20 and 25 mg mL^−1^ in 43% sulfuric acid for 30 min, and the absorbance was read at 482 nm. The standard curve was plotted based on the absorbance intensity and drug concentration and a linear equation was obtained from the standard curve. The drug-loaded meshes were placed in fresh 2 mL centrifuge tubes and 500 μL of 43% sulfuric acid was added. After 30 min of incubation, the absorbance was read at 482 nm in triplicate. The azithromycin concentration was calculated using the standard curve.

#### 2.5.3. Antibiotics Release Profile

The drug-loaded meshes were incubated in 500 μL of PBS at 37 °C with rotation, and 250 µL of PBS solution was taken for drug concentration measurement at predetermined time points: 1 h, 4 h, 1 d, 7 d and 14 d. 250 µL of fresh PBS was added to the mesh incubation tubes at each time point. Standard solutions of azithromycin (1, 5, 10, 15, 20, 25 mg/mL) were prepared by mixing drug powder in 250 µL PBS. The standard solutions were mixed with 250 µL sulfuric acid solution (43%) for 30 min. Absorbance was read at 482 nm to obtain the measurements of standard solutions and mesh samples. The standard curve was obtained using the standard solutions, and concentration of azithromycin released from the mesh samples was calculated based on the standard curve. 

### 2.6. Antimicrobial Test

The antimicrobial capacity of the mesh samples was conducted against *S. aureus* ATCC 25923 by a disk diffusion method [16]. Briefly, the bacterial strain was inoculated onto a brain heart infusion agar (Oxoid). After 24 h incubation at 37 °C, bacterial colonies were isolated and suspended in sterile saline until the turbidity was compatible with 0.5 Mac Farland. *S. aureus* suspension (100 μL) was spread onto a Mueller–Hinton agar (Oxoid) plate. The PCL and PCL/PEG meshes (5 mm) with two doses of azithromycin (1 and 0.5 mg, *n* = 3 for each dose) after 0 and 14 d of release in PBS at 37 °C were sterilized for 30 min under UV and pasted onto the agar plate and incubated for 18 h at 37 °C. Azithromycin antimicrobial susceptibility disks (15 μg, Oxoid) were used as the positive control. Unloaded meshes and mock treated meshes in DEE were used as the negative controls. The bacterial growth on the plate was visualized directly after incubation of the plates at 37 °C for 18 h, and the diameter of the inhibition zone was measured according to clinical and laboratory standards institute (CLSI M02-A10) recommendations.

### 2.7. Biocompatibility Test

The biocompatibility of the samples was tested using human immortalized adipose derived mesenchymal stem cells (hMSC) (ATCS CRC4000, ATCC). Nine MEW pelvic mesh groups were selected to test their biocompatibility: PCL_non-loaded control, PCL_drug loaded (0.5 mg azithromycin), PCL_drug released (drug-loaded meshes released for 14 days in PBS as described in 2.4.3), 90:10_non-loaded control, 90:10_ drug loaded, 90:10_ drug released; 75:25_non-loaded control, 75:25_drug loaded, 75:25_ drug released. All mesh samples were cut into disks of 5 mm in diameter and sterilized for 30 s with 70% ethanol. The mesh samples were air dried overnight in a biosafety cabinet and further sterilized with 20 min UV radiation on each side prior to cell seeding.

#### 2.7.1. Cell Seeding

The hMSC cells (passage 5) were seeded onto the mesh sample disks at a density of 1 × 10^4^ cells/disk and cultured at 37 °C, 5% CO_2_ in ATCC Mesenchymal Stem Cell Basal Medium (ATCPCS500030) supplemented with ATCC Mesenchymal Stem Cell Growth Kit (ATCPCS500040) and 0.2 mg/mL Geneticin selective antibiotics (G418 Sulphate, Thermo Fisher Scientific, Brisbane, Australia). The cells on the mesh samples were assessed for their viability and morphology with the following in vitro assays: alamarBlue assay, LIVE/DEAD assay, fluorescent microscopy and scanning electron microscopy (SEM) imaging.

#### 2.7.2. Cell Viability Assessment: LIVE/DEAD and alamarBlue Assays

The alamarBlue assay (Thermo Fisher Scientific, Brisbane, Australia) was used to quantitatively assess the cell metabolism following the manufacturer protocol. Briefly, at 1 d, 7 d and 14 d timepoints, the MSC medium was removed from 4 samples of each mesh group, and the samples were rinsed with PBS and transferred to a fresh 48-well plate. The samples were then incubated with 330 µL of fresh culture medium containing 10% (*v*/*v*) of alamarBlue solution for 4 h at 37 °C with 5% CO_2_. After the incubation, 3 aliquots of 100 µL from each sample medium were transferred to black-wall 96-well plates. The fluorescence was read at 545/590 nm (excitation/emission) with a CLARIOstar microplate reader (BMG Labtech, Mornington, Australia).

LIVE/DEAD assay was used to show the distribution of live and dead cells attached on the mesh samples. Briefly, after 1 d and 7 d of cell seeding, the mesh samples were moved to a fresh 48-well plate and washed twice with PBS solution. The disk samples were incubated for 30 min in 300 µL of LIVE/DEAD staining solution (Thermo Fisher Scientific, Brisbane, Australia) containing 2 µM calcein and 4 µM ethidium. The stained samples were imaged with a fluorescent microscope (Zeiss Axio Observer 7, Carl Zeiss, Oberkochen, Germany) immediately after the staining.

#### 2.7.3. Cell Morphology: Fluorescent Microscopy and SEM

The cell morphology stain by immunofluorescence and microscope imaging were performed as described previously [18]. Briefly, at 3 d, 7 d and 14 d timepoints, cell culture medium was removed and the mesh samples were transferred into a fresh 48-well plate. The samples were then washed in PBS and fixed in 4% paraformaldehyde (Sigma, Melbourne, Australia) for 30 min at room temperature. Following a rinse in PBS and permeabilization in 0.2% Triton X-100 solution (Sigma-Aldrich, Australia), the samples were incubated with 0.5% bovine serum albumin for 10 min. The samples were then immersed for 45 min in staining solution containing 0.8 U/mL Alexa Fluor^®^ 488 Phalloidin (Thermo Fisher Scientific, Brisbane, Australia) and 5 µg/mL 4′,6-diamino-2-phenylindole (DAPI; Thermo Fisher Scientific, Brisbane, Australia). The samples were imaged with a fluorescent microscope (Axio Observer 7, Carl Zeiss, Oberkochen, Germany).

SEM sample preparation and imaging were performed as described previously [19]. Briefly, at 3 d, 7 d and 14 d timepoints, the mesh samples were fixed in 2.5% glutaraldehyde (ProSciTech, Townsville, Australia) immediately after cell culture. The samples were washed in PBS buffer (Electron Microscopy Sciences, Hatfield, PA, USA) and dehydrated in graded ethanol solutions and dried with Hexamethyldisilazane (Sigma Aldrich, Melbourne, Australia). The gold sputter-coated samples were imaged using a TESCAN MIRA3 SEM (Tescan, Brno-Kohoutovice, Czech Republic).

### 2.8. Statistical Analysis

The statistical tests were performed by two-way ANOVA with GraphPad Prism 9 software (GraphPad Software Inc., San Diego, CA, USA). For the mechanical testing data, there was repeated violation of the assumption of normal distribution; therefore, the non-parametric Kruskal–Wallis test with post hoc analysis using Bonferroni corrected Wilcoxon signed-rank test was used. A *p* < 0.05 was considered a significant result.

## 3. Results

### 3.1. Degradation Test

**Figure 2:**
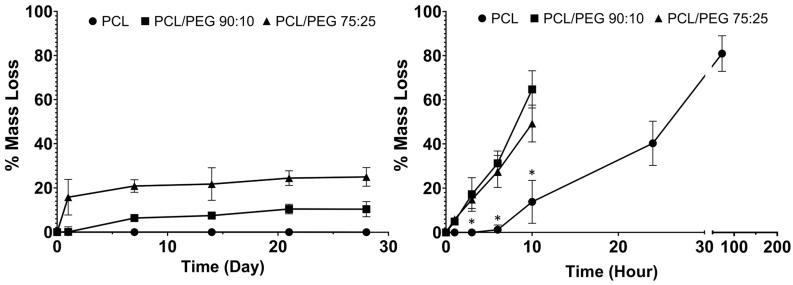
Physiological degradation (10 mL PBS at 37 °C) of PCL and PCL/PEG meshes (**left**). Accelerated degradation (10 mL 5 M NaOH at 37 °C) of PCL and PCL/PEG meshes (**right**). * shows significant less mass loss % in the PCL meshes compared to both PCL/PEG groups (*n* = 3, *p* < 0.05).

The physiological and accelerated degradation curves are shown in Figure 2, with the composite meshes shown to degrade faster than the PCL. In physiological conditions, the mass loss % increased with increasing PEG content in the mesh fibres. In the accelerated degradation test, both the 90:10 and 75:25 mesh groups showed significantly greater mass loss % compared to that of the PCL mesh group (control). The 90:10 and 75:25 groups became unretrievable after 10 h of degradation, while the PCL meshes were retrievable after 3 days in NaOH.

### 3.2. Mechanical Testing

**Figure 3:**
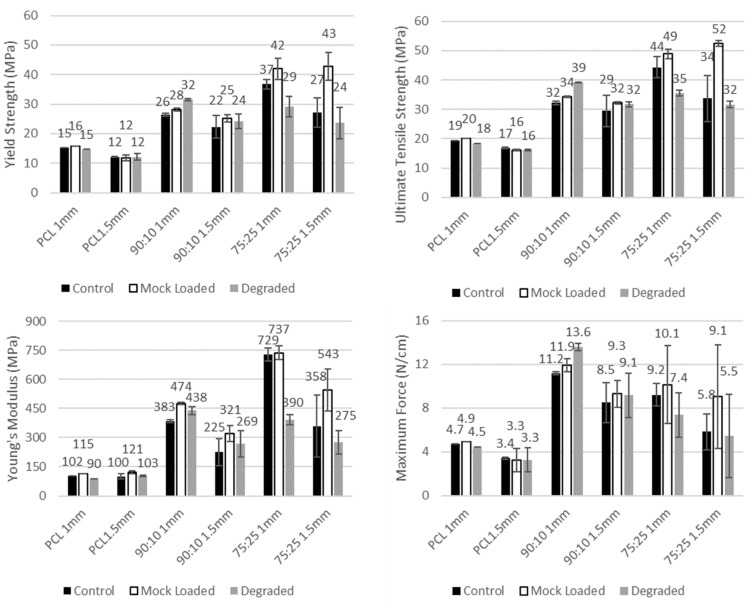
Tensile testing results, including strength (yield strength (**top left**), ultimate tensile strength (**top right**) and max force per centimetre (**bottom right**)) and stiffness (Young’s modulus (**bottom left**)) measures for each grouping. Results are given in mean with error bar denoting standard deviation.

The material composition was found to substantially affect the tensile properties of the meshes, with the composite materials (90:10 and 75:25) having consistently higher strength (yield strength UTS and maximum force) than PCL (Figure 3). The stiffness was also found to increase with increasing PEG content, with 75:25 typically having a higher Young’s modulus than 90:10, and both higher than PCL in the non-degraded state (Figure 3; a 276% increase for 90:10 over PCL and a 615% increase for 75:25 over PCL for 1.0 mm controls). However, for the 75:25 composite, the degradation in 28 days of PBS reduced the stiffness by −46% in comparison to the 1 mm 75:25 control.

The mesh geometry with the smaller mesh spacing (1.0 mm), and, therefore, more cross fibres to bear a load, was found to have a non-significant increase in tensile strength (UTS and maximum force) when compared to 1.5 mm spacing for the same material and state (*p* > 0.99). 

The degradation of the meshes in 28 days of PBS did not result in any significant reduction in the mesh UTS or yield strength for the as printed samples for any material (degraded 1.0 mm vs. control 1.0 mm, *p* > 0.97). Further, the test setup effect of drug loading via immersion in DEE for 8 h did not result in any significant changes to the mesh tensile properties for any material (mock loaded 1.0 mm vs. control 1.0 mm, *p* > 0.99).

### 3.3. Antibiotics Release

**Figure 4:**
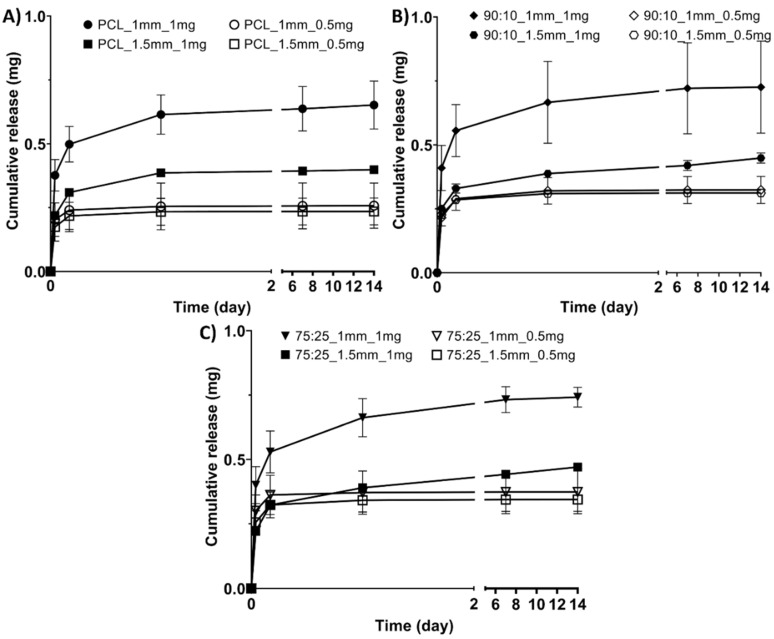
Cumulative antibiotic release profiles for the sample groups: (**A**) PCL groups, (**B**) 90:10 groups, (**C**) 75:25 groups.

Across all the materials, the mesh geometry affected the drug release, with the smaller mesh spacing (1 mm) having significantly higher cumulative antibiotics release than the larger 1.5 mm spacing (Figure 4). The 1 mg drug loaded samples had higher cumulative release than the 0.5 mg samples for all the materials (Figure 4).

### 3.4. Antimicrobial Test

**Figure 5:**
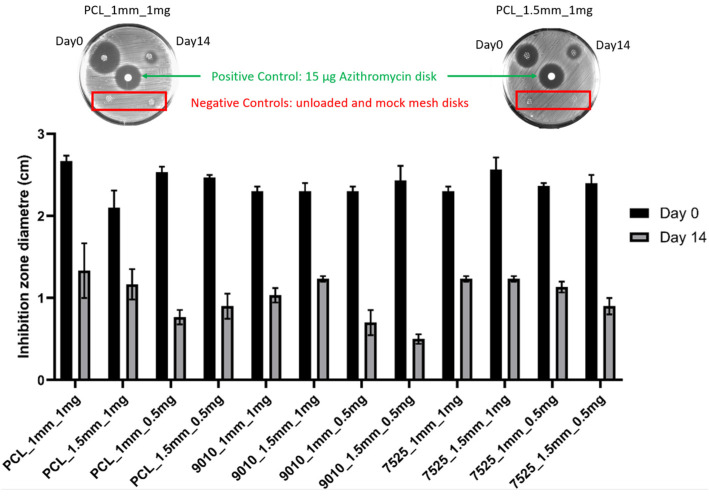
Inhibition zone measurements for day 0 and day 14 for all testing groups (**bottom**). A representative image is shown of the testing plates (**top**), where clear inhibition zones can be seen for the fabricated meshes at day 0 and day 14 and for the positive control, whereas the mock loaded and unloaded negative controls did not exhibit the inhibition zone.

Clear inhibition zones were observed for all the material groups (PCL, 90:10, 75:25) for both spacings (1.5 mm and 1.0 mm) at day 0 and day 14 (Figure 5). The 14 d measurements resulted in a significantly smaller inhibition zone compared with 0 d.

### 3.5. Biocompatibility Test

#### 3.5.1. LIVE/DEAD and alamarBlue Assay

**Figure 6:**
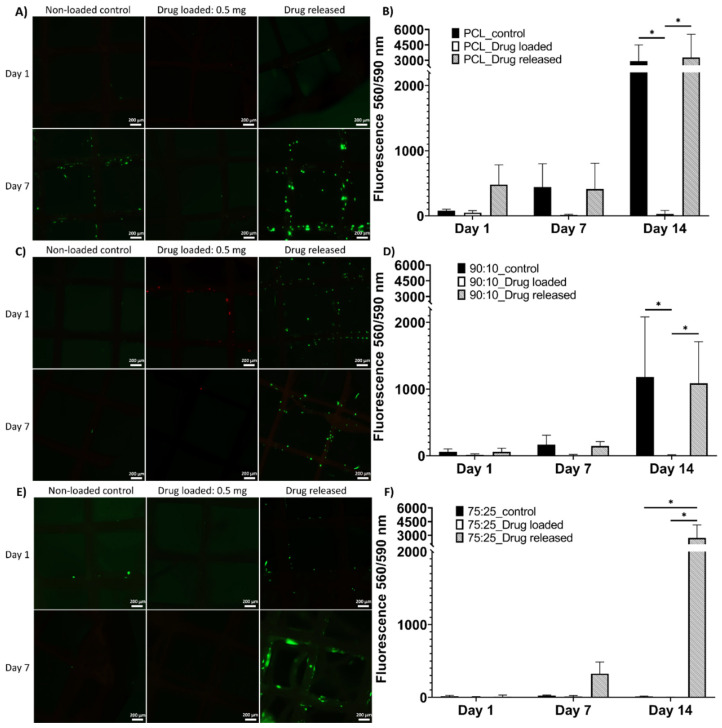
LIVE/DEAD assay and alamarBlue assay of all sample groups at all assessment time points. LIVE/DEAD assay results ((**A**) for PCL meshes, (**C**) for 90:10 meshes, (**E**) for 75:25 meshes) showing viable cells in green fluorescence. AlamarBlue results ((**B**) for PCL meshes, (**D**) for 90:10 meshes, (**F**) for 75:25 meshes) show mean± standard error mean (scale = 200 µm). In graph B and graph D, both non-loaded control group and drug released group showed significantly higher cell metabolic activity at the 14-day time point compared to the drug-loaded group. In graph F, significantly higher cell metabolic activity was found at the 14-day time point in the drug-released group compared to both the drug-loaded and non-loaded control groups. * shows statistical significance *p* < 0.05.

The LIVE/DEAD results showed the viable cells in green fluorophore and dead cells in red at 1-day and 7-day time points (Figure 6). As shown in Figure 6A, the cells were viable in the PCL non-loaded control and drug-released mesh groups; few live cells were observed in the drug-loaded group. In Figure 6C, a small number of live cells were found in the 90:10 non-loaded control group and a number of viable cells were high in the drug-released group; the cells on the drug-loaded meshes were mostly dead. In Figure 6E, a few viable cells were found on the 75:25 non-loaded control mesh, and the drug-loaded mesh samples showed almost no cell attachment. The drug-released group showed higher cell viability than the freshly drug-coated and control groups. The overall trend in the LIVE/DEAD assay showed higher numbers of viable cells at the 7-day time point, especially in the drug-released mesh groups.

The AlamarBlue assays showed cell metabolic activity that indicated cell viability and proliferation on the mesh samples at the 1-day, 7-day and 14-day time points (Figure 6). Noticeably, at the 14-day time point, a statistical difference was found between the drug-released group and drug-loaded group in all mesh materials (Figure 6B,D,F), between the control group and drug-loaded group in PCL and 90:10 materials (Figure 6B,D) and between the drug-released group and non-loaded control group in 75:25 material (Figure 6F).

#### 3.5.2. Cell Morphology by Fluorescent Microscopy

**Figure 7:**
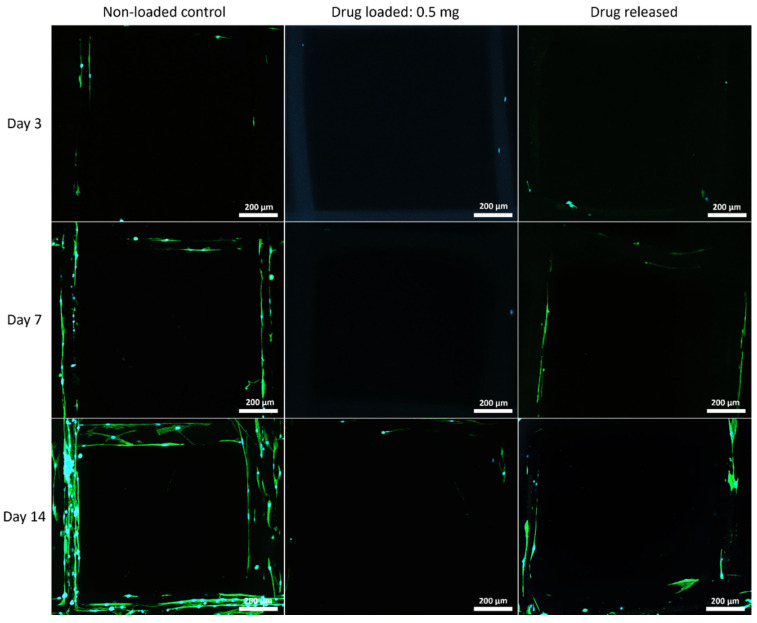
MSC cell morphology on PCL mesh groups at all time points. Cell nuclei are shown in blue and cytoskeleton shown in green.

**Figure 8:**
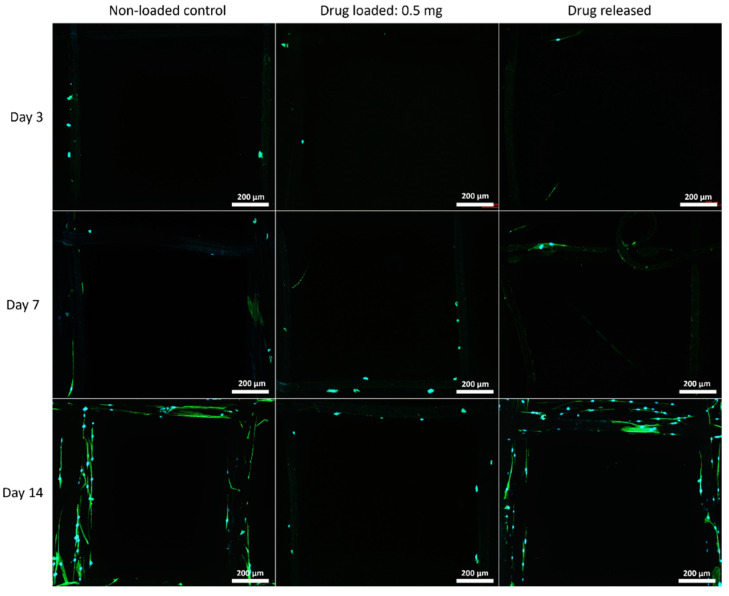
MSC cell morphology on 90:10 mesh groups at all time points. Cell nuclei are shown in blue and cytoskeleton shown in green.

**Figure 9:**
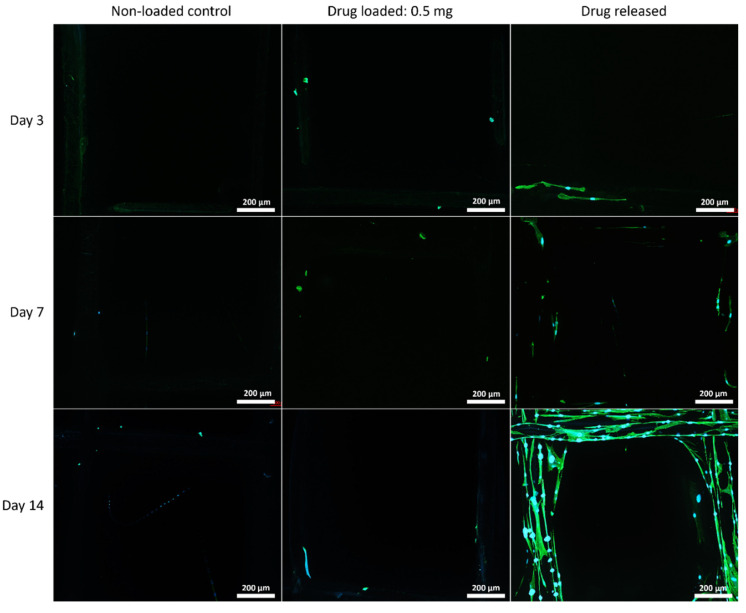
MSC cell morphology on 75:25 mesh groups at all time points. Cell nuclei are shown in blue and cytoskeleton shown in green.

The cell morphology on the mesh structures was examined with a fluorescent microscope, and the nuclei of the cells were shown in blue fluorophore and cell skeletons were shown in green (Figure 7, Figure 8 and Figure 9).

#### 3.5.3. Cell Morphology by SEM

**Figure 10:**
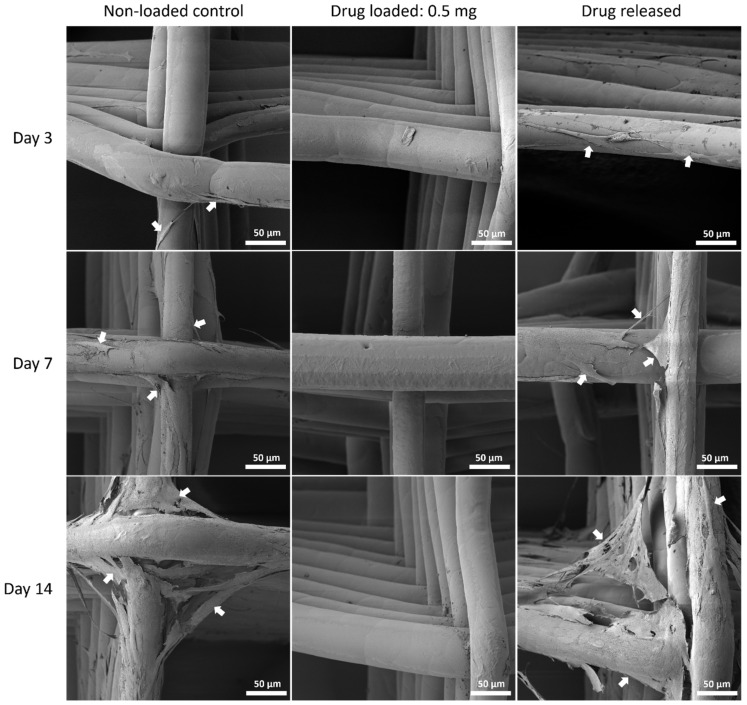
MSC cell morphology by SEM on PCL mesh groups at all time points. Arrows pointing at representative cells or cell sheets attached on mesh fibres.

**Figure 11:**
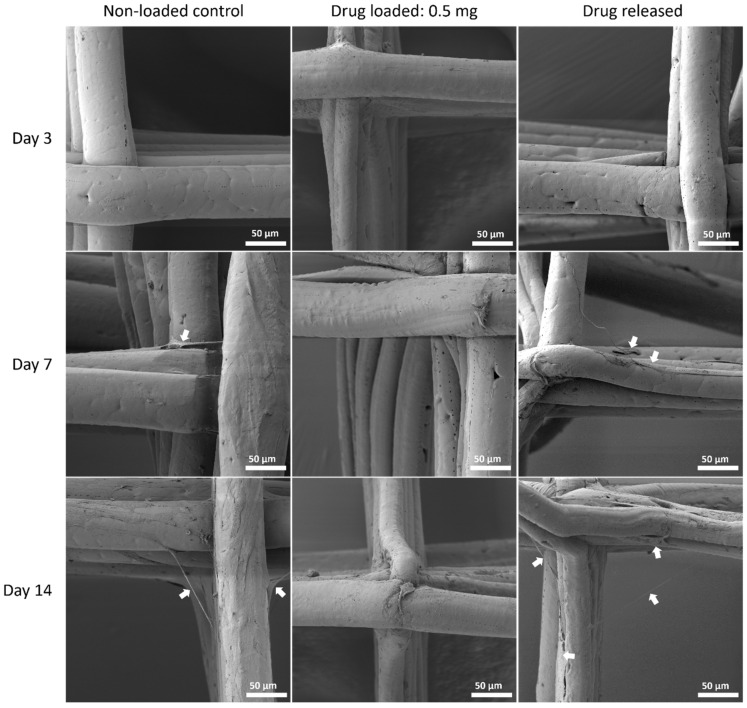
MSC cell morphology on PCL/PEG 90:10 mesh groups at all time points by SEM. Arrows pointing at representative cells or cell sheets attached on mesh fibres.

**Figure 12:**
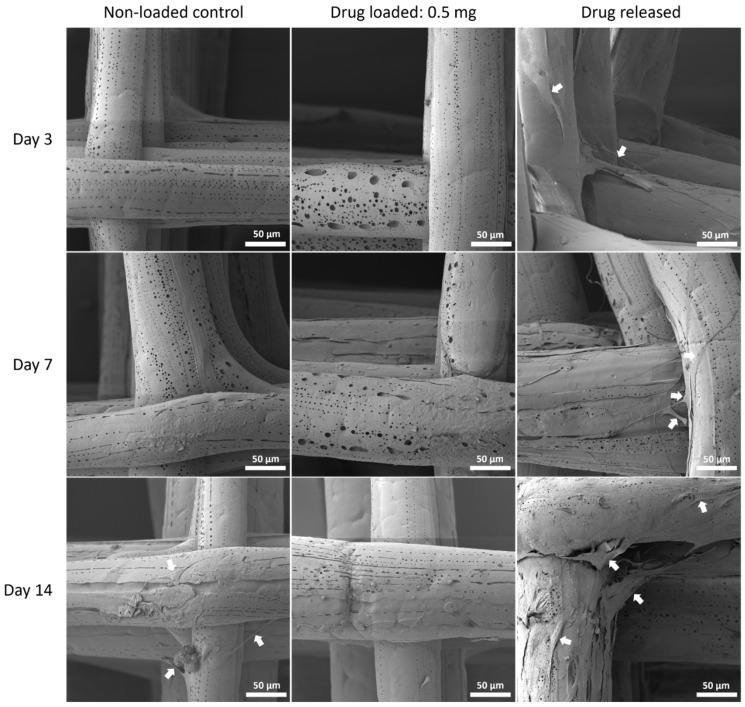
MSC cell morphology on PCL/PEG 75:25 mesh groups at all time points by SEM. Arrows pointing at representative cells or cell sheets attached on mesh fibres.

The cell morphology was assessed with SEM to show the interaction of MSC cells and the mesh surface (Figure 10, Figure 11 and Figure 12).

## 4. Discussion

The ban on pelvic mesh implants generated an urgent need for an alternate mesh product to treat POP. One of the main causes of mesh failure is the non-compliance of the polypropylene mesh. Studies have investigated methods to modify the polypropylene meshes, for example, producing different knitted mesh structures [20] or coating the mesh with electrospun polymer composite of polylactic acid (PLA) and PCL [21]. 

There has also been a growing interest in creating the pelvic meshes with biodegradable polymers such as PLA [22], PLGA [23] and PCL [24]. The use of non-degradable polymer such as polypropylene for implants tend to lead to scarring and other foreign body responses. The advantage of using biodegradable polymer such as PCL, PLA and PLGA is that they are FDA approved and widely used as biomaterials for tissue engineering purposes including pelvic meshes [25,26]. PCL, although commonly used as biomaterials, is known for its hydrophobicity which impedes cell adhesion [27]. Incorporating PEG, which is a soft, hydrophilic polymer, to create a hybrid scaffold is often a strategy employed to enhance surface hydrophilicity [28,29,30]. PEG is widely used in the field of drug delivery to produce a controlled sustainable delivery system by blending with polyurethane [31] and PCL [32,33]. PLA-PEG scaffold has also been electrospun for neural tissue engineering [30].

Electrospinning is a manufacturing technique that has gained increasing interest for fabricating pelvic meshes due to its ability to create microstructures mimicking the extracellular matrix, which has been shown to enhance tissue integration [24,26]. This present study fabricated the meshes using melt-electrowriting (MEW), a technique similar to electrospinning with the exception that MEW enables the meshes to be produced with a high degree of control and precision and does not include toxic solvents [34]. Additionally, the fibre thickness, porosity and scaffold height can be customised to produce scaffolds with optimal properties for pelvic meshes.

In this study, composites of PCL and PEG meshes with two different ratios (90:10 and 75:25 PCL:PEG) were fabricated using MEW. PEG was a good choice for the composites as it has a similar melting point to that of PCL (ΔTm ~3 °C) [35]. As medical grade PCL has a slow degradation rate (~4 years), the hydrophilic nature of PEG was utilised to alter the degradation rate of the composites. The degradation rate of PCL/PEG mesh is controllable by adjusting the content of PEG in the composite to match the rate of tissue regeneration in the pelvic floor. The degradation profile at physiological conditions (in PBS at 37 °C) showed an average of 10% mass loss in the 90:10 mesh group and ~25% mass loss in the 75:25 groups. No weight changes were observed in the PCL group (Figure 2). These results indicate that the hydrophilic PEG component was dissolved by the aqueous PBS solution and washed off from the mesh fibres, while the PCL showed no sign of degradation. Interestingly, the degraded 75:25 samples formed hollow structures, indicating the PEG component of the composite might have been more central when manufactured via MEW (Figure S1). Other PCL composites, such as PCL/PLA, showed a weight loss of less than 5% in PBS at 37 °C after 30 days, whereas PCL/PGA exhibited a loss of 55%, which would be too fast for a POP application [36]. Polypropylene, despite being a non-degradable polymer in vitro, when implanted as a mesh, underwent superficial degradation in 33% of the patients [37]. After 3 months of implantation, peeling of the fibre surface, cracks and flaking of the polymer were observed.

To examine the long-term degradation behaviour of PCL mesh and PCL/PEG composite meshes, we used 5 M NaOH for an accelerated degradation test method. The hydrophobic PCL meshes lost 80% of their mass within 3 days, while both PCL/PEG mesh groups lost their structure and became unretrievable after 10 h. At the 10-h time point, the PCL/PEG 90:10 group showed almost 5-fold increase in degradation rate and the PCL/PEG 75:25 group showed a 3.7-fold increase compared to PCL alone. Although both composite groups had a significantly faster degradation rate than PCL, interestingly, the 90:10 group degraded faster than the 75:25 group in accelerated conditions. This could be due to the change of crystallization of PCL with the increased presence of PEG when the fibres cooled down to room temperature after MEW printing [35,38]. The increased crystallinity of PCL alters its physical properties, such as increased melting temperature, degradation rate, and increased the mechanical strength with higher stiffness.

The addition of PEG to PCL increased the tensile strength of the meshes compared to the PCL control group. Increasing the PEG content to 25% exhibited an average increase in ultimate tensile strength of 99% to 34 MPa (1.5 mm) and 127% (44 MPa; 1.0 mm) in the 75:25 group). Polypropylene meshes, such as Gynemesh, exhibited tensile strength of 2.59 MPa, which is markedly lower than the PCL/PEG composites [39]. Electrospun meshes fabricated using other polymers also exhibited similar tensile strength as Gynemesh, such as PLA (3.5 MPa) and PLGA/PCL (3.6 MPa). Interestingly, PLA fibres, when aligned, produce meshes with tensile strength that increased to 22 MPa, which is similar to our PCL/PEG composites, which comprised aligned fibres [39]. Additionally, the increase in PEG also corresponded to a significant increase in stiffness (358 MPa (1.5 mm, *p* = 0.79) and 729 MPa (1 mm, *p* = 0.02) in Young’s modulus) compared to PCL alone. This increased stiffness is also likely associated with the increased crystallinity of the PCL with increased proportion of PEG. Stiffer polypropylene-based meshes, especially Gynemesh with a Young’s modulus of 9 MPa, have been shown to disrupt ECM remodelling and produce protein responses similar to vaginal degeneration [39,40]. Additionally, the stiffness of meshes can influence the rate of mesh-related complications [41,42], resulting in increasing risk of mesh exposure [40].

It is noted that there are very limited studies in the literature that utilise MEW to create meshes for POP application. Most of the studies fabricated meshes via electrospinning, producing scaffolds with lower mechanical strength than MEW scaffolds. For example, the tensile strengths of our PCL/PEG composites (~30 MPa for 90:10 group) were higher than those of other PCL composites, as shown by researchers such as Vashaghian et al. [26], whereby electrospun PCL/PLGA and PCL/Gelatin exhibited tensile strengths of 12.4 ± 1.6 MPa and 3.5 ± 0.9 MPa, respectively. The stiffness of the PVDF electrospun scaffolds ranged from 13.1 to 25.8 MPa [43] and was 10 to 20 times lower than the PCL/PEG composites. Irrespective of manufacturing techniques, the stiffness of these scaffolds was still too high when compared to premenopausal healthy vaginal tissues, which measured at 0.79 MPa [25]. On the other hand, while it is desirable to have lower mechanical properties, electrospun scaffolds have non-uniform small pore sizes, which hinders cellular infiltration and tissue integration. The ability of MEW to better control fibre thickness and pore size has the advantage of tailoring the scaffold’s parameters to obtain the desirable properties.

Although parameters such as Young’s modulus and ultimate tensile strength are commonly used to assess the mechanical characteristics of meshes, they can be difficult to compare when the mesh structures and sizes vary. Pott et al. proposed an alternative approach for mesh strength comparison by measuring maximal force that the mesh sustained over 1 cm mesh width (N/cm) [44]. As shown in Figure 3, the maximal force values of the PCL meshes with 1 and 1.5 mm spacing were 4.7 and 3.4 N/cm, respectively. Such maximal force is not adequate as the clinically relevant force for hernia repair was noted as 32 N/cm (lateral) and 22 N/cm (cranial/caudal) [44]. The PCL–PEG composite meshes improved the maximal force, whereby a 10% PEG addition exhibited an increase of 136% for the 1 mm-spaced mesh and 148% for the 1.5 mm-spaced meshes. Changes in scaffold architecture through features such as interwoven fibres, varying fibre orientation, and altering the composite composition may enable the mesh strength to approach clinically relevant levels. The pore size and shape are important to take into consideration when designing the meshes. These parameters have been shown to influence mechanical strength, in particular, the strength of tissue ingrowth. The pore sizes of commercially available meshes were wide-ranging, with 1.1 mm measured in Novasilk mesh to Ultrapro with 4 mm pore size [25]. Large pore sizes (> 1 mm) have been shown to integrate better with tissue and exhibited more tissue ingrowth in pigs [45,46]. The mechanical strength of tissue ingrowth was enhanced as the pores increased from 1 mm to 5 mm [46]. In addition, the meshes with hexagonal pores encouraged the strongest tissue ingrowth in pigs after 90 days of implantation [46]. The pore sizes of 1 and 1.5 mm of our meshes were chosen based on the literature and aimed at facilitating good integration with the host tissue.

Since the meshes aim to be ultimately implanted in humans, the biocompatibility of the various types of meshes was assessed. MSCs are commonly used in tissue engineering for cell-based therapy [47] owing to their ability to differentiate into various types of cells, such as smooth muscle cells [48] and endothelial cells [49]. MSCs also produce various types of growth factors, including VEGF, which will assist with production of blood vessels and tissue integration [50]. Additionally, MSCs are immunomodulatory, producing cytokines that regulate immune cells, such as T cells, to influence the activation of the cells involved in wound healing and tissue repair [51]. Seeding of MSCs on the PCL/PEG scaffolds has the potential of creating meshes that encourage healing and tissue integration, ultimately improving the outcome of the POP treatment. Another advantage of using MSCs for the biocompatibility test in this study is that MSCs are found to be superior to other cell types for in vitro cytotoxicity tests because they are a more accurate modelling of in vivo conditions [52].

Although PEG increased the mechanical properties of the PCL meshes, it appeared to produce a less conducive surface for cell attachment. As shown in Figure 9 and Figure 12, there were fewer MSCs observed after 14 days of culture when PEG was increased from 10% to 25%. The addition of PEG to PCL increased the hydrophilicity of the meshes, but PEG is also known to have an anti-cell attachment due to decreased initial random motility coefficient, which reduces cell-substrate adhesion in hMSC [53]. This effect is also increased with a higher content of PEG in the substrate. Endothelial cells cultured on PU/PEG composite also demonstrated initial lower cell proliferation when compared to PU scaffolds [54]. Other studies have shown that the relationship between surface wettability and cell adhesion displays a bell shape distribution rather than a linear relationship, with the ideal hydrophilicity being cell-dependent [55,56]. Polypropylene meshes cultured with endothelial cells in vitro showed a reduction in viability of almost 50% after 3 days [57], while, in vivo, they induced a proinflammation response in 27 patients, demonstrated by an increase in M1 macrophages [58].

Another complication faced by women with mesh implants is infection of the mesh. An anti-bacterial property was incorporated into the composite meshes fabricated in this study. Azithromycin was selected as the antibiotic for this study due to its broad antimicrobial coverage and previously demonstrated effectiveness in PCL fibres loaded with azithromycin [16]. Mathew et al. demonstrated that electrospun PCL fibres loaded with azithromycin via a solvent evaporation technique inhibited the growth of *Staphylococcus aureus* for 14 days [16]. Azithromycin is a widely used macrolide antibiotic that inhibits bacterial protein synthesis, which provides coverage of many Gram-positive bacteria and most Gram-negative bacteria, including ‘atypical’ bacteria, such as mycoplasma and mycobacteria species [59]. Its clinical uses include treatment of community-acquired pneumonia, otitis media, pharyngitis, cervical infections, pelvic inflammatory disease, skin infections as an alternative to beta-lactam antibiotics and as prophylaxis to vulnerable patients with advanced acquired immunodeficiency syndrome [60,61]. In this study, the antibacterial property was maintained across all three types of meshes, with the 75:25 PCL:PEG 0.5 mg azithromycin group still producing an inhibition zone of 10 mm after 14 days (Figure 5).

Although azithromycin is an effective bactericide, our study indicated that it may be cytotoxic above certain concentrations. Azithromycin has been shown to be cytotoxic to fibroblasts at a concentration of 0.05% while, at 0.025%, it was biocompatible [62]. As shown in our study by the LIVE/DEAD assay and alamarBlue assay, there were very few viable cells in the azithromycin-loaded groups across all three types of meshes throughout the 14 days of culture (Figure 6). However, in the drug-released groups, increased cell proliferation was observed across the experimental period, especially the 75:25 group (Figure 6F). As the drug released, the concentration of azithromycin on the meshes decreased gradually, thereby enabling cells to proliferate with time. This suggested that, even though there was a delay of cell attachment and proliferation when the drug-coated meshes were first incubated, the cytotoxicity decreased over time, enabling cells to eventually proliferate on the meshes. It is anticipated that the design and composition of these meshes will provide initial mechanical support to the pelvic organs and encourage tissue integration and infection-free regeneration after implantation, as demonstrated by the biocompatible and antibacterial properties of the mesh. When the regenerated connective tissues have sufficient strength to take over the mechanical load, the meshes are expected to fully degrade and be replaced by natural tissues.

Confined by the scope of the study and current understanding of biodegradable mesh usage for POP, there are limitations of this study: the laydown patterns of the MEW meshes were limited to the 90° cross hatch, only 2 PCL:PEG composites were studied and two antibiotic dosage levels were chosen. These mesh designs are substantially different from the woven, knitted and braided implants typically made of polyester, polytetrafluoroethylene, polypropylene, polyethylene and nylon [63]. Future studies will need to investigate the effect of different pore shapes to obtain mechanical properties comparable to the vaginal tissue. With better understanding of the regenerative rates of the connective tissues in the pelvic floor, PCL/PEG composite meshes can be prepared for a degradation rate that matches the tissue regeneration. Future experiments will include further optimizing the dosage of azithromycin to acquire a balance between being bactericidal and biocompatible. In vivo implantation studies in small and large animal models will also be needed to demonstrate the preclinical performance of these meshes.

In this study, altering the mesh material composition led to the greatest effect on the mesh material properties, providing evidence that mesh material properties may be tailorable via polymer composites fabricated via MEW. Careful consideration should be given to the stiffness of the designed mesh as higher stiffness meshes have influenced: the rate of mesh-related complications [41,42], tissue remodelling response through stress shielding [64,65] and breakdown of collagen and elastin [66,67], resulting in an increasing risk of mesh exposure [40].

## 5. Conclusions

This study has demonstrated that melt-electrowritten (MEW) composite meshes comprising PCL and PEG showed a controllable degradation rate by adjusting the PEG content and produced mechanical properties, such as maximal forces, that are higher than PCL alone and move towards the forces observed clinically. Antibacterial properties with slow releasing capabilities were successfully incorporated into the meshes, albeit the concentration used warrants further adjustment. A biodegradable mesh that is compliant and antibacterial appears possible to manufacture using a version of 3D printing (MEW) and would provide a much needed and urgent treatment for women with POP.

## Figures and Tables

**Table 1:** MEW parameters of PCL and PCL/PEG composite meshes.

Mesh Type	Voltage (kV)	Temperature (°C)	Tip to Collector Distance (mm)	Needle Gauge	Air Pressure (MPa)	Plate Speed
PCL/PEG 90:10	4.5	95	5	21	0.08	300
PCL/PEG 75:25	4.5	95	5	21	0.08	300
PCL (Control)	6	90	5	21	0.05	600

## Data Availability

The raw data required to reproduce these findings also form part of an ongoing study, but they are available to download on request.

## Supplementary Materials

The following supporting information can be downloaded at: https://www.mdpi.com/article/10.3390/polym14040763/s1, Figure S1. Cross sectional SEM image of a representative sample from the 75:25 degraded group (after 28 days immersion in PBS), showing the formation of a hollow structure after the PEG content was dissolved in PBS solution, indicating the PEG component of the composite might have been more central when manufactured via MEW.
