# Supplementary material for: Development of 3D Printed Biodegradable Mesh with Antimicrobial Properties for Pelvic Organ Prolapse
Source: Polymers (Basel). 2022 Feb 16;14(4):763. doi: 10.3390/polym14040763 (PMC8877663; doi:10.3390/polym14040763)
Supplement: Supplementary file 1 [file polymers-14-00763-s001.zip › polymers-1552220-supplementary.pdf]

polymers

MDPI

Article

Development of 3D Printed Biodegradable Mesh with Antimicrobial Properties for Pelvic Organ Prolapse

Jiongyu Ren [1,2], Rebecca Murray [3,4,5], Cynthia S. Wong [6], Jilong Qin [7], Michael Chen [1,2,3], Makrina Totsika [7], Andrew D. Riddell [5,8], Andrea Warwick [5], Nicholas Rukin [3,5] and Maria A. Woodruff [1,2,*]

[1] Centre for Biomedical Technologies, Queensland University of Technology (QUT), Brisbane, QLD 4000, Australia; edward.ren@qut.edu.au (J.R.); m66.chen@hdr.qut.edu.au (M.C.)
[2] School of Mechanical, Medical and Process Engineering, Queensland University of Technology (QUT), Brisbane, QLD 4000, Australia
[3] Herston Biofabrication Institute, Metro North Health, Brisbane, QLD 4029, Australia; becca.murray@health.qld.gov.au (R.M.); nicholas.rukin@health.qld.gov.au (N.R.)
[4] Australian Institute for Bioengineering and Nanotechnology, The University of Queensland, Brisbane, QLD 4072, Australia
[5] Redcliffe Hospital, Metro North Health, Redcliffe, QLD 4020, Australia; adriddell@hotmail.com (A.D.R.); mda99amw@yahoo.com (A.W.)
[6] Aikenhead Centre for Medical Discovery (ACMD), St Vincent's Hospital, Melbourne, VIC 3065, Australia; c_wong32@hotmail.com
[7] Centre for Immunology and Infection Control, School of Biomedical Sciences, Queensland University of Technology, Brisbane, QLD 4000, Australia; jilong.qin@qut.edu.au (J.Q.); makrina.totsika@qut.edu.au (M.T.)
[8] Northside Clinical Unit, School of Clinical Medicine, The University of Queensland, Brisbane, QLD 4072, Australia
* Correspondence: mia.woodruff@qut.edu.au

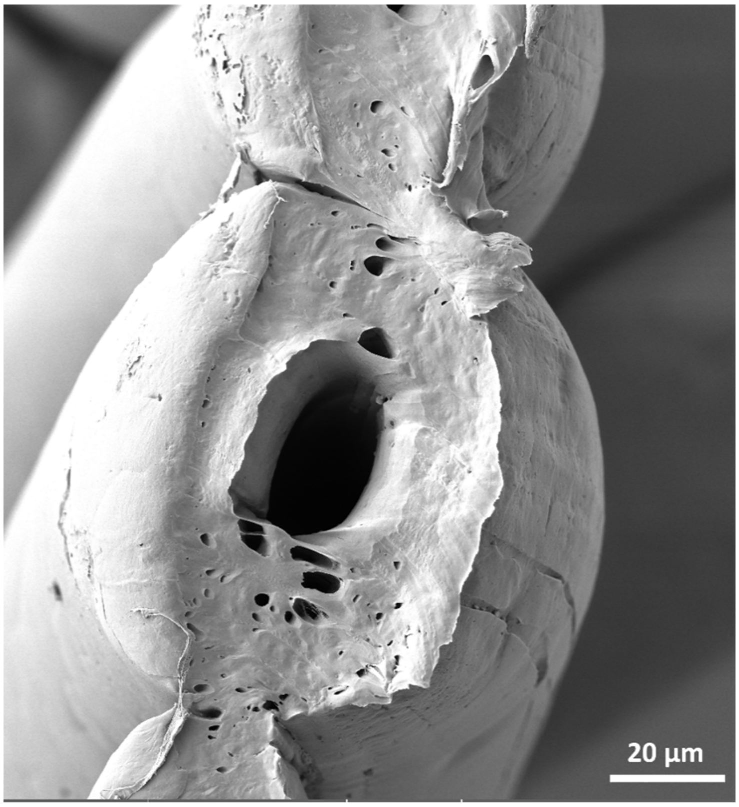

Figure S1. Cross sectional SEM image of a representative sample from the 75:25 degraded group (after 28 days immersion in PBS), showing the formation of a hollow structure after the PEG content was dissolved in PBS solution, indicating the PEG component of the composite might have been more central when manufactured via MEW.

Citation: Ren, J.; Murray, R.; Wong, C.S.; Qin, J.; Chen, M.; Totsika, M.; Riddell, A.D.; W arwick, A.; Rukin, N.; Woodruff, M.A. Development of 3D Printed Biodegradable Mesh with Antimicrobial Properties for Pelvic Organ Prolapse. *Polymers* **2022**, *14*, 763. https://doi.org/10.3390/polym14040763

Academic Editor: Matthew Blacklock

Received: 28 December 2021
Accepted: 3 February 2022
Published: 16 February 2022

Publisher's Note: MDPI stays neutral with regard to jurisdictional claims in published maps and institutional affiliations.

Copyright: © 2022 by the authors. Submitted for possible open access publication under the terms and conditions of the Creative Commons Attribution (CC BY) license (https://creativecommons.org/licenses/by/4.0/).
